# Supplementary material for: Therapeutic Potential of a Natural Blend of Aronia melancarpa, Lonicera caerulea, and Echinacea purpurea Extracts in Treating Upper Respiratory Tract Infections: Preliminary Clinical and In Vitro Immunomodulatory Insights
Source: Int J Mol Sci. 2024 Dec 15;25(24):13436. doi: 10.3390/ijms252413436 (PMC11677390; doi:10.3390/ijms252413436)
Supplement: Supplementary file 1 [file ijms-25-13436-s001.zip › ijms-3369186-supplementary.pdf]

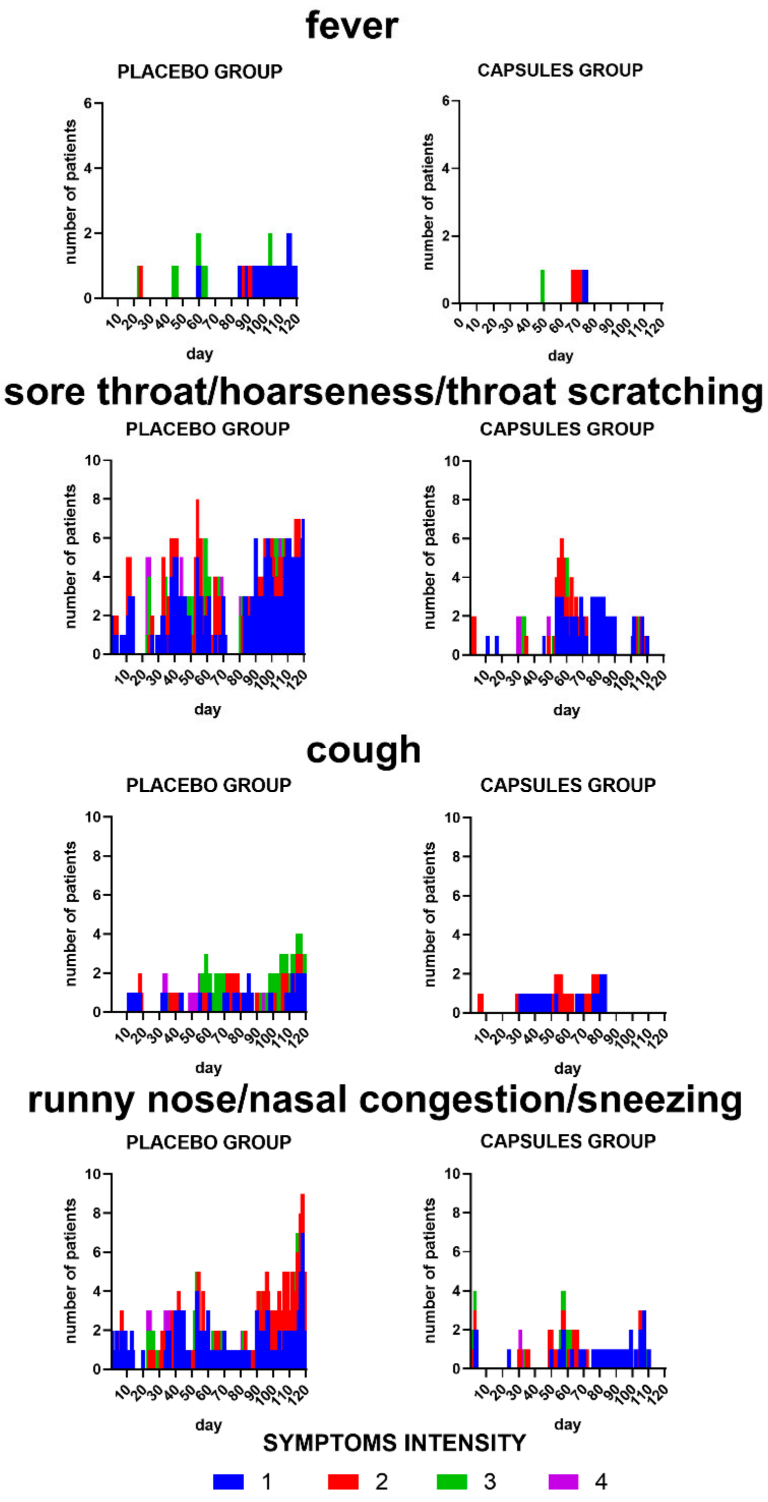

Figure S1 Daily counts of symptoms (fever, sore throat/hoarseness/throat scratching, cough, runny nose/nasal congestion/sneezing) from Day 1 to Day 120, categorized by severity (from 1 to 4) and group (placebo vs. capsules). This figure illustrates the frequency and intensity of each symptom as reported on the day of occurrence, comparing outcomes between the treatment groups.

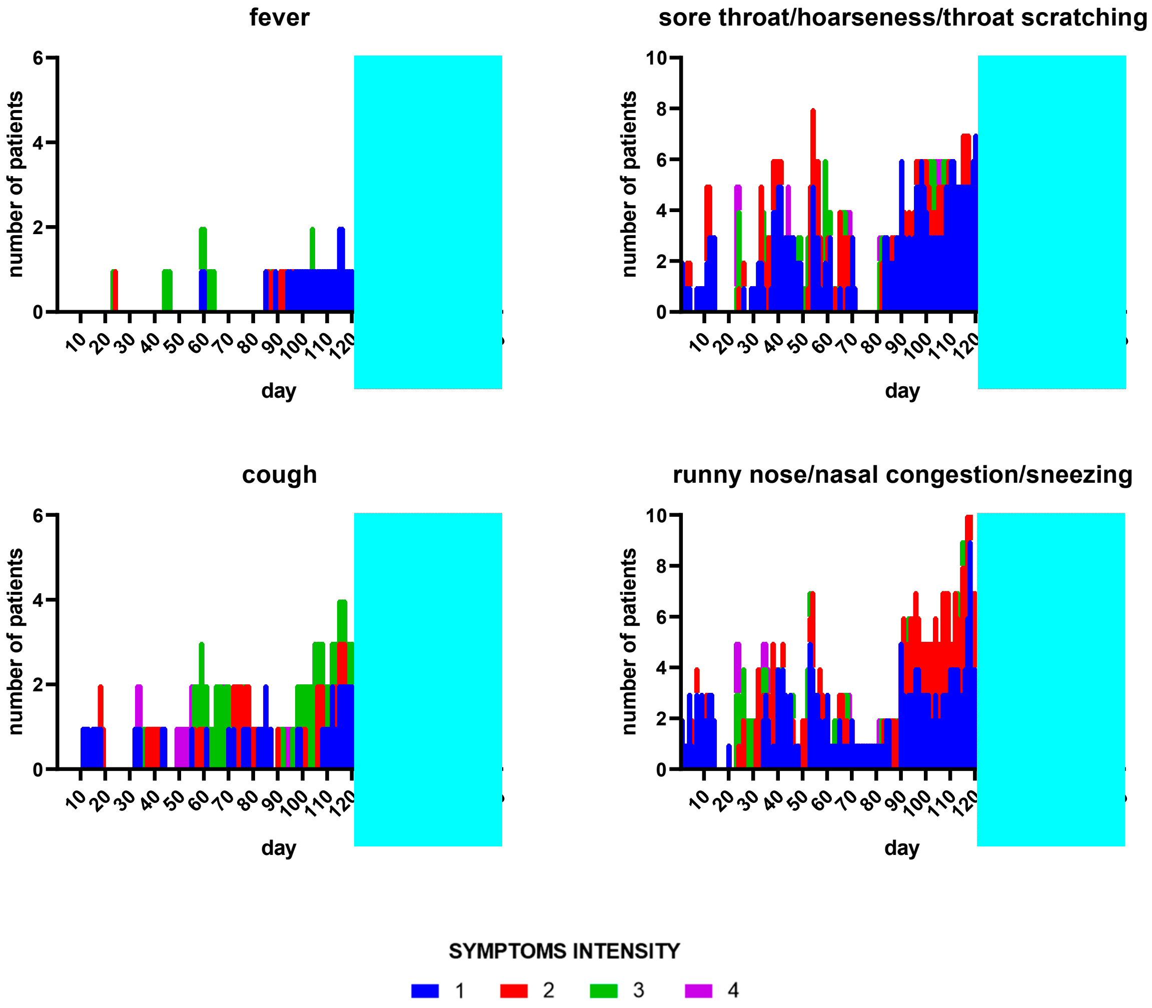

Figure S2 Daily counts of symptoms (fever, sore throat/hoarseness/throat scratching, cough, runny nose/nasal congestion/sneezing) from Day 1 to Day 180, categorized by severity (from 1 to 4). This figure illustrates the frequency and intensity of each symptom as reported on the day of occurrence, detailing the progression within the same cohort initially under placebo treatment from D1-D120 and subsequently during syrup supplementation from D121-D180. The period of syrup supplementation is indicated in light blue.

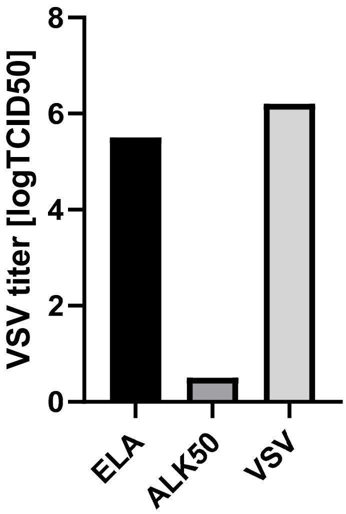

Figure S3 The antiviral activity of M3.The graph shows the average of 3 independent experiments (n=3). ALK50 - positive control; VSV - negative control.

Table S1 The impact of the ELA blend on immune system indicators in patients treated with the ELA blend in both capsule and syrup forms. The dataset includes measurements of serum CRP concentration (C-Reactive Protein). SOD activity (Superoxide Dismutase), and the production of cytokines such as IFN-α (Interferon-alpha), IL (Interleukin)-6, IL-1β, and TNF-α (Tumor Necrosis Factor-alpha) in PBMCs (Peripheral Blood Mononuclear Cells) stimulated with PHA-L (Phytohemagglutinin-L) or R848 (Resiquimod). Statistical evaluations are represented through Median (Me), Median of Differences (MEDIAN OF DIFF.), Interquartile Range (IQR), Confidence Interval (CI), and p-values (p).

**SAMPLE**	**PARAMETER**	**GROUP**	**BEFORE**		**AFTER**		**BEFORE VS AFTER**		
			**Me**	**IQR**	**Me**	**IQR**	**p**[1]	**MEDIAN OF DIFF.**	**CI**
**serum**	**CRP [mg/L]**	**SYRUP + CAPSULES**	1.1	2.0	1.4	2.0	***0.045***	0.085	-0.01 to 0.33
	**SOD [units/mL]**	**SYRUP + CAPSULES**	62.2	76.4	62.3	56.4	*0.766*	-0.65	-5.5 to 2.9
**supernatant from PBMCs stimulated with R848**	**IFN-α [pg/mL]**	**SYRUP + CAPSULES**	97.0	133.8	116.3	103.9	*0.721*	3.9	-11.9 to 21.9
**supernatant from PBMCs stimulated with PHA-L**	**IL-6 [pg/mL]**	**SYRUP + CAPSULES**	406.8	513.9	513.8	499.4	*0.303*	71.5	-17.6 to 210.3
	**IL-1β [pg/mL]**	**SYRUP + CAPSULES**	12.1	13.5	14.3	26.6	***0.018***	5.4	-2.0 to 11.2
	**TNF-α [pg/mL]**	**SYRUP + CAPSULES**	170.5	211.5	196.3	148.5	***0.015***	66	20.4 to 82.5

[1]*p value for Wilcoxon test*
