# Supplementary material for: Therapeutic Potential of a Natural Blend of Aronia melancarpa, Lonicera caerulea, and Echinacea purpurea Extracts in Treating Upper Respiratory Tract Infections: Preliminary Clinical and In Vitro Immunomodulatory Insights
Source: Int J Mol Sci. 2024 Dec 15;25(24):13436. doi: 10.3390/ijms252413436 (PMC11677390; doi:10.3390/ijms252413436)
Supplement: Supplementary file 1 [file ijms-25-13436-s001.zip › Patient diary.pdf]

Study number	
Patient Number	

Participant Diary

You have received a diary to complete during the study period (either electronically or on paper, depending on your preference). If you are filling out the paper version, please use BLOCK CAPITAL LETTERS and initial each page. If you have any questions, please contact the study investigator. In case of concerning symptoms, please call – [phone numbers].

Check sign

Study number	
Patient Number	

Supplement Use:

Day	Date	The number of supplement tablets taken each day	Any additional medications used (not specified in the Case Report Form, such as paracetamol for headaches)	Additional symptoms (e.g., headache, cold, fever)		
				What happened?		
				When?	Duration	Intensity
D0						
D1						
D2						
D3						
D4						
D5						
D6						
D						
D						
D						

Check sign

Study number	
Patient Number	

Disease Symptom Questionnaire:

Please indicate the symptoms/disease signs that occurred during the day. The severity of the symptoms should be rated on a scale from 0 to 4, where: 0 = No symptoms, 1 = Mild symptoms, 2 = Moderate symptoms, 3 = Severe symptoms, 4 = Very severe symptoms. Enter the appropriate numerical value for each symptom. If symptoms other than those listed are observed, describe the symptom in detail and assign it a severity score as well.

**Symptom**	Observation Day/Date						
Runny nose/nasal discharge							
Nasal congestion/mucosal swelling							
Fever (temp. >38°C)							
Sore throat							
Hoarseness							
Throat irritation							
Cough							
Weakness							
Sleepiness							
Muscle pain							
Headache							
Sneezing							
Abdominal pain							
Nausea							
Vomiting							
Diarrhea							
Constipation							
Breathing difficulty							
Rash							
Dry mouth							
Other symptoms (please specify): Other symptoms (please specify):							

Check sign

Study number	
Patient Number	

Check sign
